# Alteration of the immune microenvironment in the axillary metastatic lymph nodes of luminal A breast cancer patients

**DOI:** 10.1186/s12957-024-03454-x

**Published:** 2024-06-27

**Authors:** Min Wu, Shuo Wang, Keyu Yuan, Bingjun Xiong, Yanping Li, Shuzhen Lyu

**Affiliations:** 1 Department of Breast Surgery, Beijing Shijitan Hospital, Capital Medical University, Tieyi Road 10, Haidian District, Beijing, 100038 China; 2 Department of Medical Oncology, Beijing Key Laboratory for Therapeutic Cancer Vaccines, Capital Medical University Cancer Center, Beijing Shijitan Hospital, Beijing, 100038 China

**Keywords:** Breast cancer, Luminal A, Immune function, Alteration, Positive rate

## Abstract

**Background:**

The alteration of the immune microenvironment in the axillary metastatic lymph nodes of luminal A breast cancer patients is still unclear.

**Methods:**

Postsurgical tissues from the enrolled luminal A BCs were divided into five categories: primary BC lesion at stage N0 (PL1), primary BC lesion at stage N1 (PL2), negative axillary lymph node at stage N0 BC (LN1), negative axillary lymph node at stage N1 BC (LN2), and positive axillary lymph node at stage N1 BC (LN3). The frequencies of positive immune markers (CD4, CD8, PD1, PD-L1, T-cell immunoglobulin and mucin domain 3 (TIM3), and forkhead box protein 3 (Foxp3)) in the above tissues were quantified by AKOYA Opal Polaris 7 Color Manual IHC Detection Kit.

**Results:**

A total of 50 female patients with luminal A BC were enrolled in this study. Among these patients, 23 had stage N1 disease, and 27 had stage N0 disease. Compared with that in the PL2 subgroup, the frequency of PD-1-positive cells was significantly greater in the PL1 subgroup, whether at the stromal or intratumoral level (P value < 0.05). Both the frequency of CD8 + T cells in LN1 and that in LN2 were significantly greater than that in LN3 (P value < 0.05). The frequency of TIM3 + T cells in LN1 was significantly greater than that in PL1 (P value < 0.05). The frequency of CD8 + TIM3 + T cells was significantly greater in both the LN2 and LN3 groups than in the PL2 group (P value < 0.05). The frequency of CD4 + Foxp3 + T cells was significantly greater in LN1 than in PL1 (P value < 0.05), which was the same for both LN3 and PL2 (P value < 0.05).

**Conclusion:**

Increased frequencies of CD8 + PD1+, CD8 + TIM3 + and CD4 + Foxp3 + T cells might inhibit the immune microenvironment of axillary metastatic lymph nodes in luminal A breast cancer patients and subsequently promote lymph node metastasis.

## Introduction

In recent years, breast cancer has become the most common malignancy worldwide, accounting for 30% of all new tumour cases and 15% of all deaths due to cancer. The World Health Organization revealed that the number of global breast cancer deaths will increase to 13.1 million by 2030, and breast cancer is currently the second leading cause of cancer deaths in women worldwide. Although the incidence of breast cancer in China is lower than that in countries such as Europe and the United States, since 1990, the incidence of breast cancer in China has increased twice as fast as the world average, especially in urban areas. The mortality rate of Chinese women with breast cancer is significantly greater than that of women in Europe and the United States [1]. A recent study reported that the 5-year survival rate of Chinese women with breast cancer was 73% [2], while the 5-year survival rate of American women with breast cancer was 90%. Thus, Chinese women have special pathological characteristics of breast cancer, and there is an urgent need for improved clinical treatment.

Breast cancer is a highly heterogeneous tumour, and molecular staging has gradually replaced tumour-node-metastasis (TNM) staging for guiding clinical treatment, assessing treatment efficacy and predicting the prognosis of breast cancer patients [3]. Based on the levels of oestrogen receptor (ER), progesterone receptor (PR), human epidermal growth factor receptor 2 (Her-2) and Ki-67, breast cancer was classified into four molecular subtypes: luminal A (ER + and PR ≥ 20%, Her-2-, Ki-67 < 20%); luminal B (ER + and/or PR+, Her-2-, Ki-67 ≥ 20%), or (ER + and/or PR+, Her-2+); Her-2 overexpression (ER-,PR-, Her-2+); and triple-negative (ER-,PR-, Her 2-) [4]. Luminal A breast cancer accounts for almost half of new breast malignancies and is the most common molecular subtype of breast cancer [5].

Luminal A breast cancer has a high degree of cell differentiation and a low risk of local and regional recurrence and distant metastasis, with a relatively good prognosis [3]. Ipsilateral axillary lymph node metastasis is one of the most important routes of breast cancer metastasis, and approximately half of primary breast cancer patients have ipsilateral axillary lymph node metastasis [6]. Among the 1386 patients with breast cancer in our hospital, 395 had luminal A breast cancer, accounting for 28.5% of all breast cancer patients; the proportion of patients with axillary lymph node metastasis at the time of diagnosis was 26.3%, among which the incidence of axillary lymph node metastasis was 12.9% in patients with a primary tumour ≥ 2 cm (T1). Metastasis to draining lymph nodes is an important step in the progression of breast cancer and is an important predictor of patient prognosis and survival. The anterior lymph node is the first station lymph node in which breast cancer drains, the first site of tumour-specific T-cell activation, and the most direct site of tumour-mediated immunosuppression [7]. Zuckerman et al. reported that immune-related signals in metastatic lymph nodes in patients with breast cancer were downregulated, while other pathways, such as cell cycle, DNA repair, ubiquitin and tumour-promoting signals, were upregulated, which suggested that immune cells in metastatic lymph nodes are reduced or dysfunctional compared to those in nonmetastatic lymph nodes [8]. In patients with gastric cancer, regulatory T-cell pressure populations are significantly greater in draining lymph nodes [9]. Similarly, the number of peri-cancerous CD8 + T cells was reduced in metastatic lymph nodes compared to uninvolved regional lymph nodes in patients with head and neck tumours, suggesting a local downregulation of cellular immunity [10]. In addition, the occurrence of lymph node metastasis in prostate cancer patients is associated with decreased immune responsiveness [11]. However, alterations in the T-cell populations in metastatic axillary lymph nodes in breast cancer patients are unclear, especially in patients with the most frequent type of luminal A breast cancer.

## Materials and methods

### Clinical population

The study population included female patients who underwent mastectomy at Beijing Shijitan Hospital, Capital Medical University and were pathologically diagnosed with luminal A breast cancer from September 2020 to July 2022. In this study, luminal A breast cancer was defined as an ER-positive, PR-positive (≥ 20%), Her-2-negative or Ki-67 < 20% based on immunohistochemistry and fluorescence in situ hybridization (FISH) results. Luminal B breast cancer patients were defined as patients with ER + and/or PR + status, Her-2- status, and Ki-67 expression ≥ 20% according to immunohistochemistry and FISH. Luminal A BC and luminal B BC are also classified as luminal BC. This study was approved by the institutional review board of Beijing Shijitan Hospital, and informed consent was obtained from all enrolled patients.

The inclusion criteria were as follows: (1) female breast cancer patients hospitalized in our hospital, (2) pathological diagnosis of primary luminal A breast cancer (ER + and PR-positive ≥ 20%, Her-2-, Ki-67 < 20%, detected by using immunohistochemistry and FISH), and (3) not receiving neoadjuvant therapy before surgery. The exclusion criteria were as follows: (1) patients previously treated with any form of immunotherapy, (2) patients with concomitant autoimmune disease, and (3) patients who were unable to participate in the study due to various medical conditions.

Postsurgical tissues from the enrolled patients were obtained and divided into five categories: primary BC lesion at stage N0 (PL1), primary BC lesion at stage N1 (PL2), negative axillary lymph node at stage N0 BC (LN1), negative axillary lymph node at stage N1 BC (LN2), and positive axillary lymph node at stage N1 BC (LN3).

The relevant clinical information, including sex, age, levels of ER, PR, Her-2 and Ki-67, histological grade, tumour size, vascular cancer thrombus, axillary lymph node status and Her-2 FISH results, was obtained through pathology reports.

### Testing of the immune microenvironment

The included tissues were taken from luminal A patients, and tested by antibodies of CD4 (ZM0418 antibody), CD8 (666868-1-1G antibody), PD1 (ZM0318 antibody), PD-L1 (AB237726 antibody), TIM3 (AB241332 antibody) and Foxp3 (AB20034 antibody) to observe frequencies of T-lymphocyte subsets using AKOYA Opal Polaris 7 Color Manual IHC Detection Kit (REF: NEL811001KT). The data were collected by multispectral imaging (Vectra 3, Akoya Biosciences, USA), and the results were statistically analysed to compare the effect of the immune microenvironment on the occurrence of axillary lymph node metastasis between the above groups.

### Statistical analysis

Differences in the ratios of T-lymphocyte subsets between luminal A breast cancers with or without axillary lymph node metastasis were assessed using the Mann‒Whitney U test or *t* test, and a *P* value < 0.05 was considered to indicate statistical significance. The multicolour immunohistochemical data are expressed as the mean ± standard deviation. The sections were analysed using SPSS software and GraphPad Prism software.

## Results

### Characteristics of the included patients

**Fig. 1:**
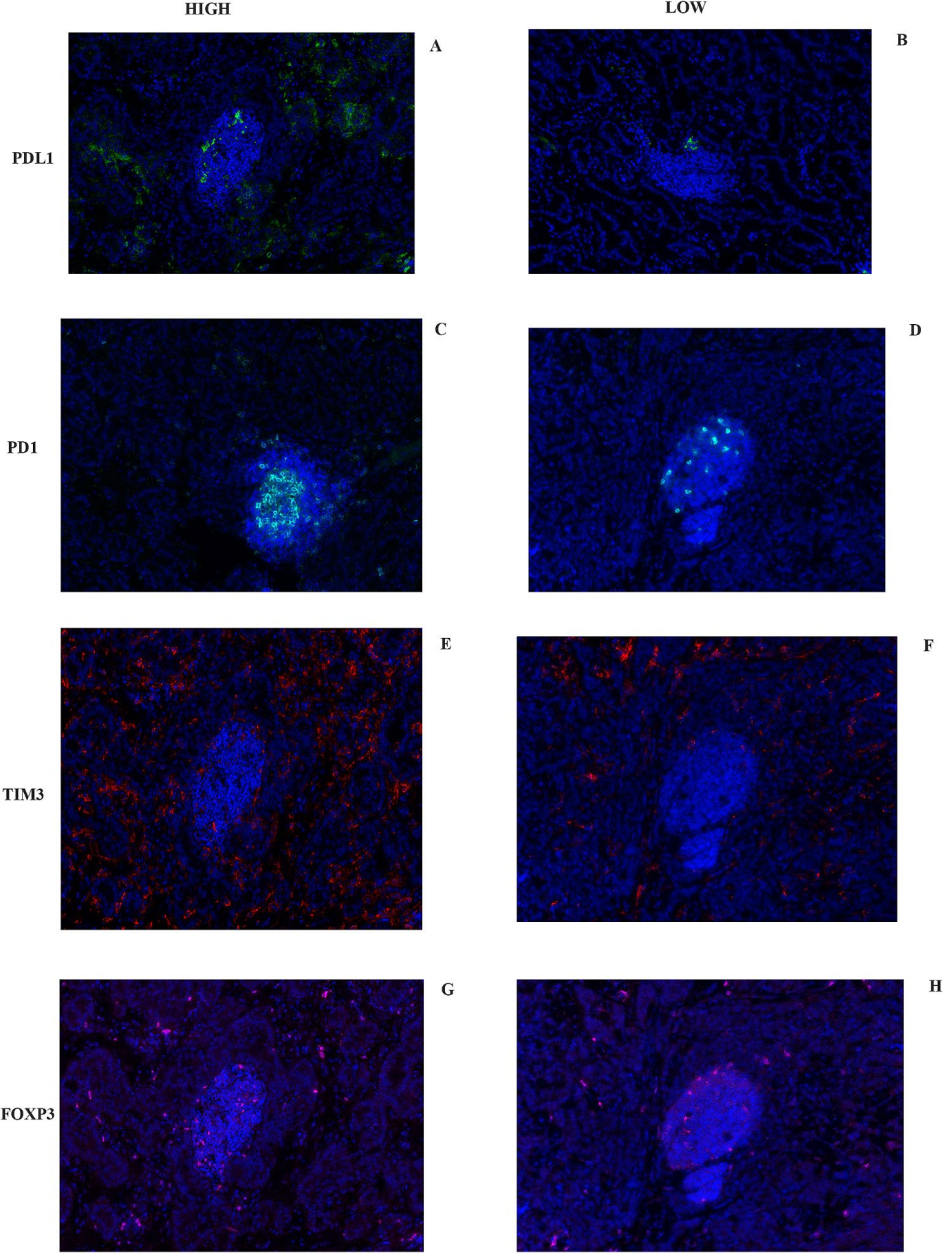
Typical images of immune markers (PDL1/PD1, TIM3, and Foxp3) in metastatic axillary lymph nodes of patients with luminal A BC. **High**: **A**, **C**, **E**, **G**. **LOW**: **B**, **D**, **F**, **H**

**Fig. 2:**
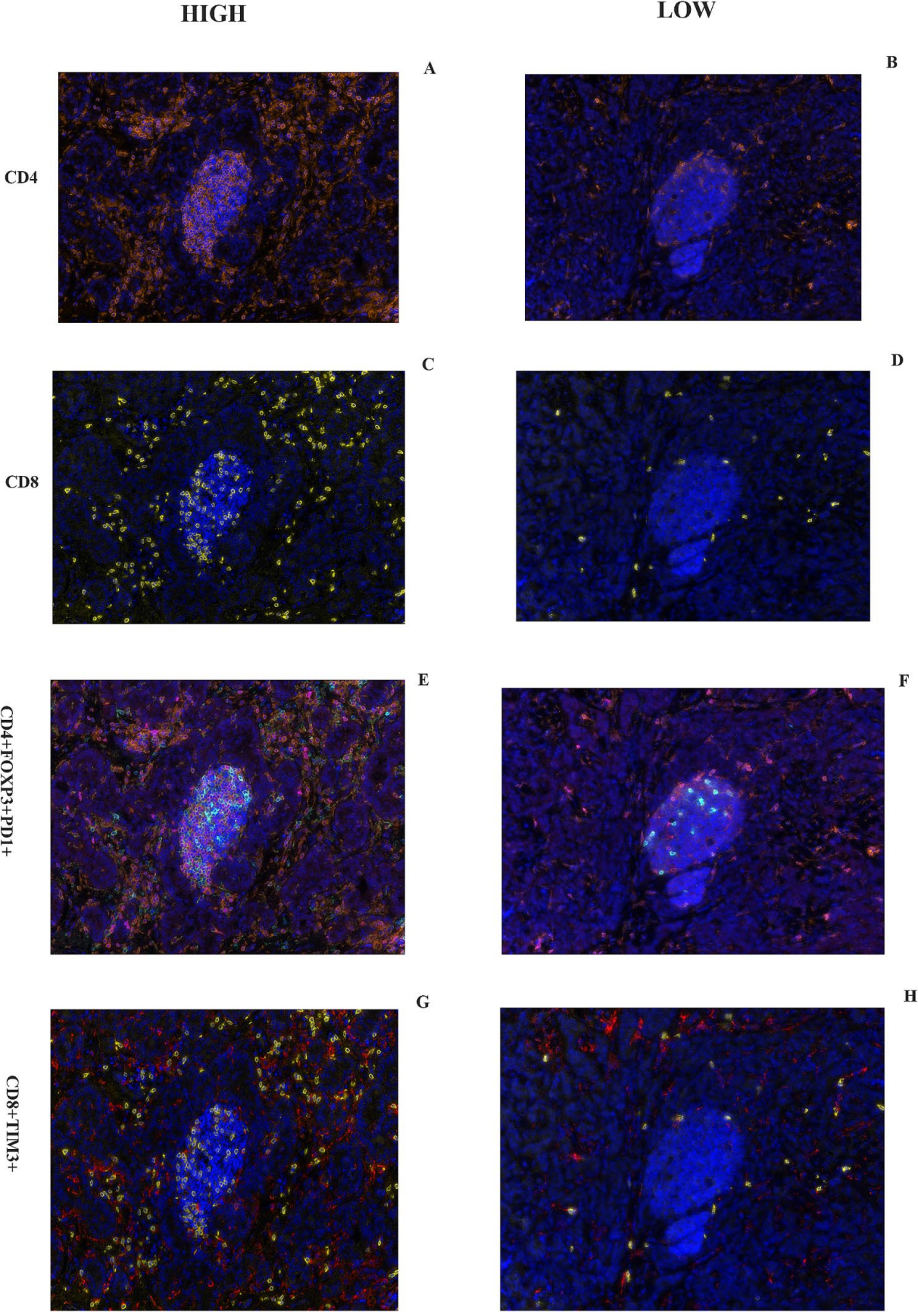
Typical images of immune markers (CD4, CD8, CD4 + Foxp3 + PD1+, and CD8 + TIM3+) in metastatic axillary lymph nodes from patients with luminal A BC. **High**: **A**, **C**, **E**, **G**. **LOW**: **B**, **D**, **F**, **H**

A total of 50 female patients with luminal A BC were enrolled in this study. 46% of patients were diagnosed with axillary lymph node metastasis. 62% of patients had a tumour size less than 2 cm. The other details of the clinical characteristics are presented in Table 1. Typical images of immune markers in metastatic axillary lymph nodes from patients with luminal A BC are presented in Figs. 1 and 2.

**Table 1:** The clinic characteristics of the included patients with Luminal A breast cancer

Item	Number of cases (percent)
Female	50
Age (year)	
≦45	8 (16)
45–65	29 (58)
≧ 65	13 (26)
Menopause	
Yes	14 (28)
No	36 (72)
Tumor size (cm)	
≦ 2	31 (62)
>2	19 (38)
Grade	
1	9 (18)
2	40 (80)
3	1 (2)
Vascular invasion	
Yes	15 (30)
No	35 (70)
Perineural invasion	
Yes	16 (32)
No	34 (68)
Axillary lymph node metastasis	
Yes	23 (46)
No	27 (54)
pTNM	
I	15 (30)
II	22 (44)
III	13 (26)

### Positivity rate for PDL1/PD1 in tissues from patients with luminal A BC

**Fig. 3:**
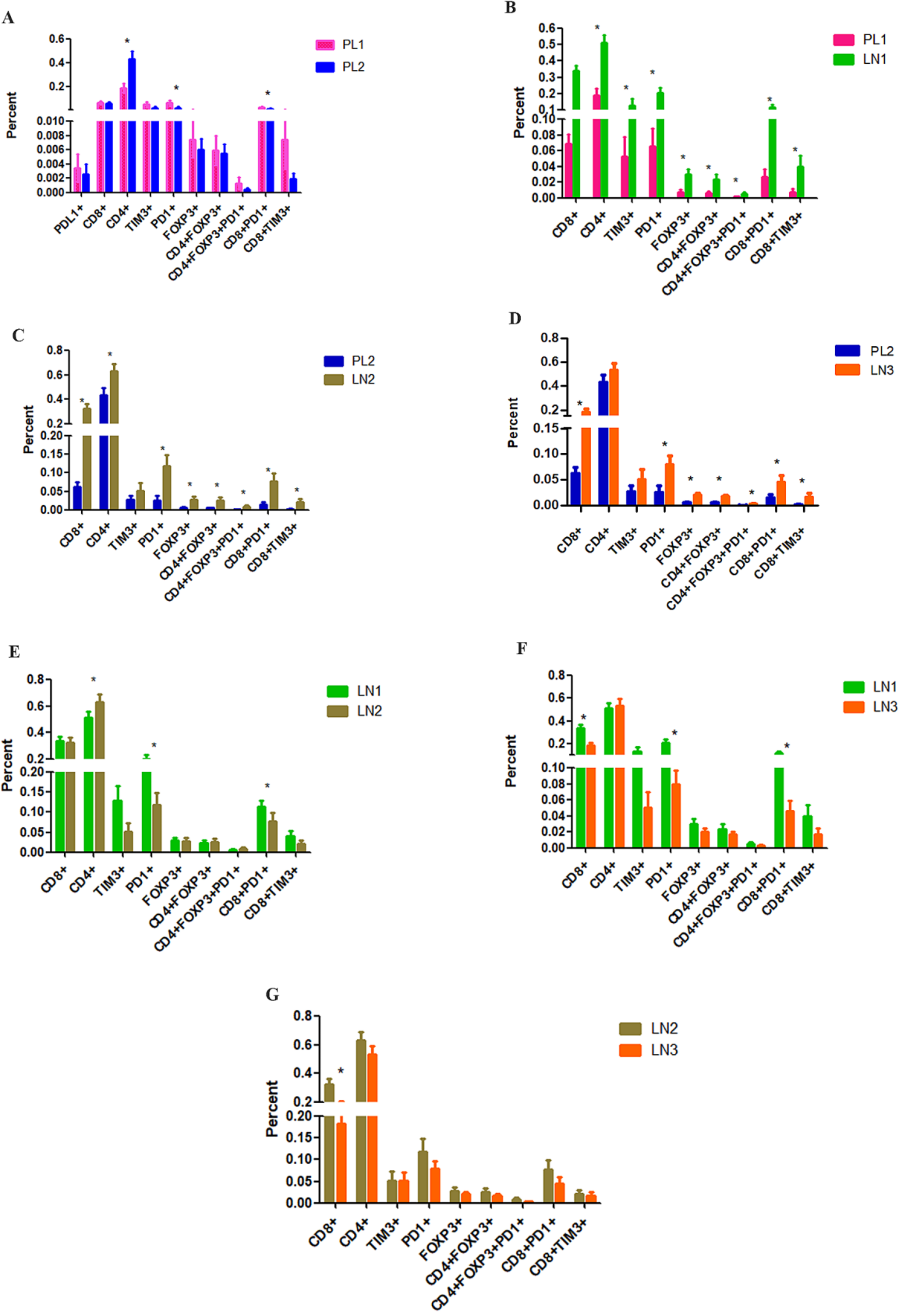
The frequency of PDL1/PD1, TIM3, CD8, CD4 and Foxp3-positive cells in tissues from patients with luminal A BC. **A**. PL1 versus PL2. **B**. PL1 versus LN1. **C**. PL2 versus LN2. **D**. PL2 versus LN3. **E**. LN1 versus LN2. **F**. LN1 versus LN3. **G**. LN2 versus LN3. BC: breast cancer, PL1: primary BC lesion at stage N0, PL2: primary BC lesion at stage N1, LN1: negative axillary lymph node at stage N0 BC, LN2: negative axillary lymph node at stage N1 BC, and LN3: positive axillary lymph node at stage N1 BC. *: *P* < 0.05

**Fig. 4:**
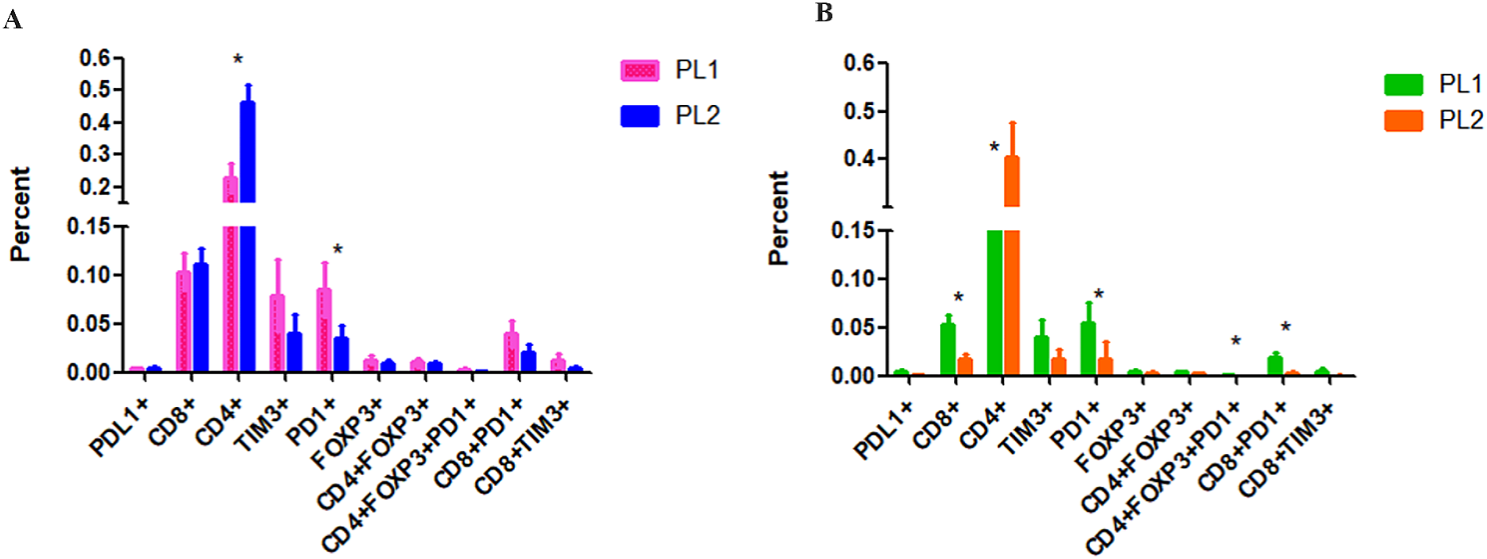
The stromal and intratumoral frequencies of tumour infiltrating lymphocytes (PDL1/PD1, TIM3, CD8+, CD4 + and Foxp3+) in primary lesions from patients with luminal A BC. **A**. the difference of stromal tumour infiltrating lymphocytes between PL1 and PL2. **B**. the difference of intratumoral tumour infiltrating lymphocytes between PL1 and PL2. BC: breast cancer, PL1: primary BC lesion at stage N0, PL2: primary BC lesion at stage N1, LN1: negative axillary lymph node at stage N0 BC, LN2: negative axillary lymph node at stage N1 BC, LN3: positive axillary lymph node at stage N1 BC. *: *P* < 0.05

The frequencies of patients with positive PDL1/PD1 expression in the immune microenvironment of luminal A BC tissues and axillary lymph nodes with or without metastasis were quantified. The frequency of PD1-positive cells was significantly greater in the PL1 subgroup than in the PL2 subgroup (*P* < 0.05) (Fig. 3A); in the stromal cell subgroup, this difference was more significant (*P* < 0.05) (Fig. 4A). However, neither the total nor the stromal frequency of PDL1-positive cells was significantly altered in BC patients with or without metastasis (*P* < 0.05) (Figs. 3A and 4A and B).

Compared with that in the PL2 subgroup, the frequency of PD1-positive cells was significantly greater in the PL1 subgroup, both in the stromal and intratumoral level (*P* < 0.05) (Fig. 4A and B). The frequency of PD1-positive cells was significantly greater in the LN1 subgroup than in the PL1 subgroup (*P* < 0.05) (Fig. 3B). A similar result was also observed between LN2 and PL2 (Fig. 3C). The frequency of PD1-positive cells was significantly greater in LN3 than in PL2 (*P* < 0.05) (Fig. 3D).

Compared with those in LN2 and LN3, the frequency of PD1-positive cells was significantly greater in LN1 (*P* < 0.05) (Fig. 3E-F). However, compared with that in LN2, the frequency of PD1-positive cells in LN3 was not significantly different (*P* > 0.05) (Fig. 3G).

### Positive rate of TIM3 expression in tissues from patients with luminal A BC

The frequency of TIM3 + T cells in LN1 was significantly greater than that in PL1 (*P* < 0.05) (Fig. 3B). Moreover, the frequency of CD8 + TIM3 + T cells was significantly greater in both the LN2 and LN3 groups than in the PL2 group (*P* < 0.05) (Fig. 3C and D). However, under other conditions, the frequency of TIM3-positive cells was not significantly different (*P* > 0.05).

### Positivity rates of the T-cell subsets in tissues with luminal A BC

The frequency of CD4 + T cells was significantly greater in the PL2 group than in the PL1 group (*P* < 0.05) (Figs. 3A and 4A and B). However, compared with those of PL2 patients, the intratumoral frequency of CD8 + and CD8 + PD1 + T cells was significantly greater (*P* < 0.05) (Figs. 3A and 4A and B). The frequency of CD4 + Foxp3 + T cells was significantly greater in LN1 than in PL1 (*P* < 0.05) (Fig. 3B).

The frequency of CD4 + Foxp3 + T cells was also significantly greater in the LN3 group than in the PL2 group (*P* < 0.05) (Fig. 3B). The frequency of CD4 + T cells was significantly greater in LN2 than in LN1 (*P* < 0.05) (Fig. 3E). Furthermore, either the frequency of CD8 + T cells in LN1 or that in LN2 was significantly greater than that in LN3 (*P* < 0.05) (Fig. 3F-G). Compared to those in LN2 and LN3, the frequency of CD8 + PD1 + T cells was greater in LN1 (*P* < 0.05) (Fig. 3F-G). Except for the frequency of CD8 + T cells, there were no significant differences in the number of sub-lymphocytes among the observation indicators between LN2 and LN3 (*P* > 0.05) (Fig. 3F-G).

## Discussion

In this study, we observed changes in immune T lymphocytes in luminal A BC patients with or without metastatic lymph nodes and differences in tumour-infiltrating lymphocytes in primary lesions. We found that in contrast to those in primary luminal A BC lesions with or without metastasis, the total frequencies of CD8 + PD1+, CD8 + TIM3 + and CD4 + Foxp3 + T cells were significantly greater in axillary lymph nodes with or without metastasis. However, compared to the number of lymph nodes with or without metastasis in stage N1 luminal A BC patients, the frequency of CD8 + PD1 + cells was significantly greater in axillary lymph nodes in stage N0 luminal A BC patients.

Tumour-infiltrating lymphocytes (TILs) are an important component of the immune microenvironment and include several subpopulations, such as CD8 + cytotoxic T cells, CD4 + helper T cells, and CD20 + B cells. Studies have shown that a high frequency of the above sub-TILs is associated with better survival [12, 13]. In this study, we observed a higher frequency of intratumoral CD4 + T cells and a lower frequency of intratumoral CD8 + T cells in primary BC at stage N1 than in BC without metastasis. In contrast to our results, node-positive luminal A BCs were reported to be associated with increased numbers of Treg cells and a decreased CD8+/Treg ratio [14]. Furthermore, a high frequency of Foxp3 + regulatory T cells (Tregs) relative to CD8 + T cells might be related to reduced progression-free survival and overall survival in breast cancer patients [15]. In contrast, this study did not observe differences in Foxp3 + Tregs between primary luminal A BC patients at stage N1 and stage N0. The immunosuppressive microenvironment of breast tumours also includes molecular checkpoints that can block antitumour immunity, one of which is programmed cell death protein-1/programmed death-ligand 1 (PD1/PD-L1). PD-L1 binds to the PD1 receptor on T cells, decreasing the antitumour activity of T lymphocytes and promoting immune escape [16]. Tumour cells may also counteract the activated antitumour immune response by upregulating PD-L1 expression through a program known as adaptive immune resistance [17]. Multiple studies have shown that PD-L1 expression on IBC tumour cells is correlated with a lack of ER expression, an increased number of TILs, a response to chemotherapy, and a triple-negative phenotype [18, 19]. Studies have shown that high PD-L1 expression combined with high CD8 + density is associated with poor prognosis in early breast cancer patients [20]. However, in this study, we did not observe differences in PDL1 expression between primary luminal A BC patients at stage N1 and stage N0. Some articles have reported that CD8 + PD1 + T cells kill tumour cells in vitro but have an inhibitory effect on tumour cells in vivo due to the action of PD-L1 [21]. Similarly, in this study, compared with that of primary luminal A BC at stage N1, the intratumoral frequency of CD8 + PD1 + T cells was significantly greater in primary BC at stage N0.

Takada et al. reported that breast cancer patients with a low density of tumour-infiltrating lymphocytes were more likely to develop axillary lymph node metastasis [22], which was consistent with the results of the present study. However, the role of functional T cells in metastatic versus nonmetastatic lymph nodes has not been well studied, and the immune effects in metastatic lymph nodes on the primary tumour are unclear. Compared with those of tumour-uninvolved (N-) nodes in lung cancer, the frequencies of CD3 + CD8 + and CD3 + CD8 + TIM-3 + T cells are lower, and the frequency of CD3 + CD8 + PD-1 + T cells is greater among tumour-involved (N+) nodes [23]. We also observed a similar phenomenon in the lymph nodes of patients with BC. However, we did not observe a difference in the frequency of CD8 + TIM-3 + T cells in the metastatic or nonmetastatic lymph nodes of patients with BC. This finding is also different from the results of Shariati S’s study, which reported that the CD8 + TIM-3 + T-cell frequency was correlated with the number of metastatic lymph nodes in BC [24]. The reason for this may be that this study focused on luminal A BC, and different detection methods were used.

Other investigations have shown that in addition to suppressing breast tumours, T cells might facilitate the progression of BC through immune surveillance or the expression of growth factors [25]. Consistently, we also showed that compared with those in primary BC lesions, the frequencies of negative regulatory lymphocytes, such as CD8 + PD1 + lymphocytes, CD8 + TIM3 + lymphocytes and CD4 + Foxp3 + lymphocytes, were increased in draining lymph nodes. We speculate that this may promote lymph node metastasis in luminal A BC patients. Consistent with our results, the frequency of CD8 + TIM3 + T cells, which can mediate antitumour immunity, was greater in patients with lymph node metastasis of invasive ductal carcinoma [26]. Furthermore, CD4 + Foxp3 + T cells in breast tumour-draining lymph nodes can highly express tumour necrosis factor receptor 2, an immunosuppressive factor [27].

In conclusion, this study investigated the immune status of lymph nodes in luminal A BCs at stage N0 and stage N1 and the differences in tumour-infiltrating lymphocytes between primary BCs at stage N0 and stage N1. This study might provide a basis for further exploration of the mechanism of breast cancer axillary lymph node metastasis. However, more investigations are still needed to explore the underlying mechanisms of immune alterations in the tumour-draining lymph nodes of luminal A BCs.

## Data Availability

No datasets were generated or analysed during the current study.

## Abbreviations

BC: Breast Cancer
TNM stage: Tumour-Node-Metastasis stage
ER: Estrogen receptor
PR: Progesterone receptor
Her-2: Human epidermal growth factor receptor 2
FISH: Fluorescence in situ hybridization
PL1: Primary BC lesion at Stage N0
PL2: Primary BC lesion at Stage N1
LN1: Negative axillary lymph node at Stage N0 BC
LN2: Negative axillary lymph node at Stage N1 BC
LN3: Positive axillary lymph node at Stage N1 BC
TIL: Tumour infiltrating lymphocytes
TIM3: T-cell immunoglobulin and mucin domain 3
Foxp3: Forkhead box protein 3
PD1/PD-L1: Programmed cell death protein-1/programmed death-ligand 1
Treg: Regulatory T cell
